# The clinical efficacy and safety of sodium-glucose co-transporter 2 inhibitors (SGLT2i) in patients with acute myocardial infarction: a meta-analysis of randomized controlled trials

**DOI:** 10.1097/MS9.0000000000003724

**Published:** 2025-09-05

**Authors:** Muhammad Sami Khan, Tallal Mushtaq Hashmi, Hanzala Jehangir, Muhammad Faiq Akram, Irja Munawar, Sanjna Devi Jagani, Maham Maqsood, Ayesha Khalid, Maryam Ijaz, Usman Saeed, Uzair Jafar, Muhammad Ehsan, Lawrence Sena Tuglo, Wajeeh Ur Rehman, Mouhamed Amr Sabouni, Nabil Braiteh, Alon Yarkoni, Keyoor Patel

**Affiliations:** a Department of Medicine, Calderdale and Huddersfield NHS Foundation Trust, Huddersfield, UK; b Department of Medicine, Rawalpindi Medical University, Rawalpindi, Pakistan; c Department of Medicine, Sheikh Zayed Medical College, Rahimyar Khan, Pakistan; d Department of Medicine, Allama Iqbal Medical College, Lahore, Pakistan; e Department of Medicine, Jinnah Sindh Medical University, Karachi, Pakistan; f Department of Medicine, Liaquat National Hospital and Medical College, Karachi, Pakistan; g Department of Cardiology, King Edward Medical University, Lahore, Pakistan; h Department of Nutrition and Dietetics, School of Allied Health Sciences, University of Health and Allied Sciences, Ho, Ghana; i Department of Internal Medicine, United Health Services Hospital, Johnson City, NY, USA; j Department of Cardiovascular Disease, University of Alabama at Birmingham, Birmingham, AL, USA; k Department of Cardiology, Mercy One Siouxland Heart and Vascular Center, Sioux City, IA, USA; l UHS Heart & Vascular Institute, United Health Services Hospital, Johnson City, NY, USA

**Keywords:** acute myocardial infarction, AMI, dapagliflozin, empagliflozin, SGLT2 inhibitors, sodium-glucose co-transporter 2 inhibitors

## Abstract

**Objective:**

Sodium-glucose co-transporter 2 (SGLT2) inhibitors improve cardiovascular outcomes in patients with heart failure (HF), but the evidence of their efficacy in patients who have had an acute myocardial infarction (AMI) is still incompletely established. This review aimed to assess the safety and efficacy of SGLT2 inhibitors on cardiovascular and structural outcomes in patients who had a recent AMI, irrespective of HF.

**Methods:**

We searched various electronic databases, including MEDLINE (via PubMed), Embase, the Cochrane Library, and ClinicalTrials.gov, till February 2025 to retrieve randomized controlled trials comparing SGLT2 inhibitors to placebo in patients with AMI. We performed a statistical analysis on RevMan 5.4 using the random effect model.

**Results:**

Our meta-analysis included seven RCTs involving 11 302 patients compared SGLT2 inhibitors to placebo in patients with AMI. SGLT2 inhibitors significantly decreased the rate of hospitalization for HF (RR 0.73, 95% CI: 0.61-0.88) with no significant change in mortality (RR 1.05, 95% CI: 0.78-1.40), all-cause hospitalization (RR 1.00, 95% CI: 0.84-1.17), and cardiovascular death (RR 1.03, 95% CI: 0.83-1.28). The incidence of hepatic injury, ketoacidosis, hypoglycemia, or lower limb amputation remained comparable across the two groups. SGLT2 inhibitors did not cause a significant reduction in N-terminal pro–B-type natriuretic peptide (NT-pro BNP) from baseline (MD −0.28 95%, CI: −0.61-0.05) nor improved the left ventricular ejection fraction at follow-up (MD 0.62, 95%, CI −0.73-1.97) compared to the placebo.

**Conclusion:**

In conclusion, while SGLT2 inhibitors show promise in reducing hospitalization for HF post-AMI, their impact on mortality and safety outcomes necessitates further investigation. This underscores the need for larger, more diverse RCTs to fully illustrate their role and timing of initiation in AMI management. An individualized approach based on risk assessment should guide their use in the post-AMI population.

## Introduction and background

Sodium-glucose co-transporter 2 (SGLT2) inhibitors are a popular group of antidiabetic drugs well known for their cardiac and renal protective effects^[1]^. SGLT2 inhibitors inhibit proximal tubular reabsorption of glucose, leading to glucosuria and enhanced diuresis^[2]^. Patients who have suffered an acute myocardial infarction (AMI) are more likely to experience heart failure (HF) or death, especially if they exhibit left ventricular systolic dysfunction^[3]^. Studies indicate that 15-25% of individuals experiencing an AMI develop HF within a year, which increases the risk of death by at least 3- to 4-fold^[3-5]^. Therefore, there remains a significant unmet need for novel therapeutic strategies within this population.

Recently, there has been success in using SGLT2 inhibitors to treat HF, leading to a decrease in cardiovascular deaths and length of hospital stay regardless of diabetes or ejection fraction status^[6-9]^. They have also been shown to be efficacious in reducing mortality in chronic kidney disease and patients with type 2 diabetes with atherosclerotic cardiovascular disease^[10,11]^. However, the use of SGLT2 inhibitors in AMI remains elusive^[12]^. Given their broad cardiorenal protective effects, investigating SGLT2 inhibitors’ role in AMI patients represents a critical next step in optimizing post-infarction care^[13]^. Currently, patients with AMI are administered several evidence-based interventions shortly after the infarction to reduce the risk of overt cardiac remodeling, HF, sudden cardiac death, and end-stage heart disease^[11,14]^. Despite the substantial progress achieved, there remains a compelling need to identify additional evidence-based strategies, as contemporary rates of adverse cardiovascular outcomes remain unacceptably high^[15,16]^.

The potential advantageous impact of SGLT2 inhibitors on postinfarction remodeling is mediated through diverse molecular mechanisms, such as the suppression of oxidative stress levels and the inhibition of cardiac inflammatory pathways^[17]^. In animal studies, SGLT2 inhibitors in AMI have been shown to reduce no-reflow and preserve cardiac function by preventing endothelial damage^[18]^. Recently, large randomized controlled trials in both diabetic and nondiabetic populations have pointed toward the potential advantage of SGLT2 inhibitor therapy in AMI^[13]^. Among the SGLT2 inhibitors, empagliflozin and dapagliflozin are included in the recent European and American guidelines for HF management^[19]^.

Given the recent body of evidence, it becomes imperative to investigate the use of SGLT2 inhibitors in AMI. Although there are meta-analyses on the effect of SGLT2 inhibitors on cardiometabolic markers after myocardial infarction, there is a lack of data on the impact of SGLT2 inhibitors on clinical and functional cardiac remodeling outcomes in patients with AMI^[20–22]^. Recently, three large-scale trials addressing the effects of SGLT2 inhibitors on cardiometabolic and structural parameters in patients with AMI have been published^[23–25]^. This meta-analysis was conducted to bridge the outlined gap by investigating the efficacy and safety of SGLT2 inhibitor therapy on clinical and cardiac remodeling outcomes in patients with AMI irrespective of HF.

## Materials and methods

This systematic review and meta-analysis were registered with PROSPERO (CRD42024550492) and conducted following the guidelines outlined in the Cochrane Handbook for Systematic Reviews of Interventions and reported according to the Preferred Reporting Items for Systematic Reviews and Meta-Analysis (PRISMA) statement (Supplementary Digital content, Table S1, available at: http://links.lww.com/MS9/A928)^[26,27]^. A TITAN guidelines checklist was followed^[28]^. This meta-analysis does not include any patient-collected/investigated data directly by the authors, so formal ethical approval was not necessary.

**HIGHLIGHTS**

- The clinical efficacy of sodium-glucose co-transporter 2 inhibitors (SGLT2i) in patients with acute myocardial infarction remains unknown.
- SGLT2 inhibitors are associated with a reduction in hospitalization for heart failure.
- The association is not affected by the type of SGLT2i used.
- No significant change in cardiovascular and all-cause mortality.

### Data sources and searches

**Figure 1.**
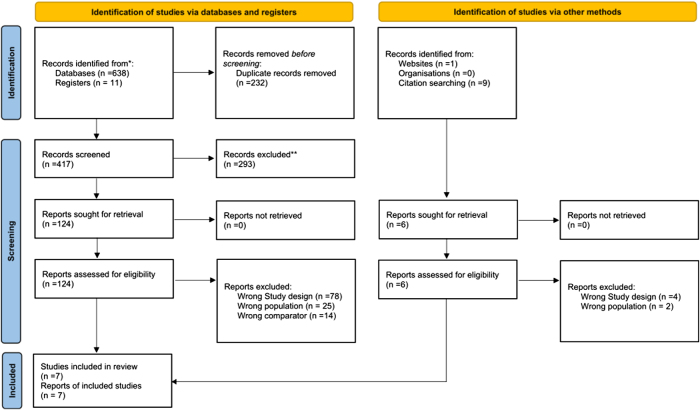
PRISMA flowchart for the screening process.

We searched the following databases and registered trials from inception till February 2025: Cochrane Central Register of Controlled Trials, MEDLINE, PUBMED, and ClinicalTrials.gov using a search strategy consisting of relevant strategy and Medical Subject Headings (MeSH) (Supplementary Digital content, Table S2, available at: http://links.lww.com/JS9/F19). We also scouted the grey literature sources such as Google Scholar and did backward citation tracking of the included articles to identify relevant articles further. The detailed search strategy is given in Fig. 1.

### Eligibility criteria

Our inclusion criteria comprised the following PICOS: (1) Study design: RCTs; (2) Population: patients with AMI irrespective of HF; (3) Intervention: Any SGLT2 inhibitor irrespective of the dosage; (4) Comparator: placebo; (5) Outcome: reporting at least one outcome of interest.

The exclusion criteria were as follows: (1) all study designs other than RCTs, such as quasi-randomized trials and observational studies; (2) studies that assessed the prophylactic role of SGLT2 inhibitors before the AMI; (3) studies conducted on animals.

### Selection process

We used the Rayyan for screening and deduplication of all the studies retrieved through our online search. Following deduplication, two authors independently screened all the titles and abstracts. This was followed by a comprehensive full-text screening by the same authors. Any disagreement between the two authors was settled by a third author.

### Outcomes

Our primary outcomes comprised hospitalization for HF and all-cause mortality.

The secondary outcomes included cardiovascular death, all-cause hospitalization, N-terminal pro–B-type natriuretic peptide (NT-pro BNP) difference from baseline, left ventricular ejection fraction (LVEF) at follow-up, hepatic injury, hypoglycemia, ketoacidosis, and lower limb amputation.

### Risk of bias assessment

For the assessment of the internal validity of included RCTs, two authors independently used the revised Cochrane “Risk of Bias” tool (RoB 2.0) which assesses bias in the following 5 domains: (1) bias in the randomization process; (2) bias due to deviations from intended interventions; (3) bias arising by missing outcome data; (4) bias occurring in the measurement of the outcome, and (5) bias in the selection of the results reported. The risk of bias was categorized for each included study as low, high, or some concerns. Any disagreements between the two authors were resolved by a third reviewer.

### Data synthesis

We run the meta-analysis on the intention-to-treat basis on Review Manager (RevMan, Version 5.4; The Cochrane Collaboration, Copenhagen, Denmark) under a random-effects model utilizing risk ratio (RR) and mean difference (MD) with corresponding 95% confidence intervals (CIs) as the effect measures. *I*^2^ > 50% was considered to indicate substantial heterogeneity. We did a subgroup analysis based on the type of SGLT2 inhibitor for the primary outcomes. Finally, all the data were compiled using trial sequential analysis. We planned to assess publication bias using a funnel plot, provided the number of studies was more than 10 in an analysis.

## Results

### Study selection and characteristics of included studies

Following our comprehensive search and analysis strategy, we identified seven RCTs that met our inclusion criteria, involving a total of 11 302 patients^[23,24,29–32]^. The detailed selection process is outlined in the PRISMA flowchart (Fig. 1). Two RCTs were multinational^[23,24]^ while the other five were from one country each, including the United Kingdom^[25]^, Japan^[31]^, Sweden^[32]^, Austria^[30]^, and Egypt^[29]^. Five trials used empagliflozin^[24,30–32]^ while two used dapagliflozin^[23,29]^. Empagliflozin was used orally as a 10 mg formulation in four RCTs^[24,25,30,31]^ and 25 mg in one RCT^[32]^, while dapagliflozin was used as 10 mg orally in two RCT^[23,29]^. Two studies (SOCOGAMI and EMBODY)^[31,32]^ were limited to diabetic patients, DAPAMI^[23]^ and DACAMI^[29]^ included only nondiabetic patients, while the EMPACTMI^[24]^, EMPRESSMI^[25]^, and EMMY^[30]^ trials included patients irrespective of their diabetic status. The detailed characteristics of trials and patients are given in Table 1.

**Table 1:** Summary of characteristics of individual trials

Study ID	Location	Sample size	Age^b^	Male, *n* (%)	BMI kg/m^2^(%)	Hypertension *n* (%)	Type 2 diabetes *n* (%)	Intervention group	Control group	Clinical outcomes reported by trial	Follow-up duration
Butler *et al* 2024	Multiple c	6522 (3260 vs 3262)	63.6 ± 11.0 vs 63.7 ± 10.8	2448 (75.1) vs 2449 (75.1)	28.1 (5.0) Vs 28.1 (5.0)	2262 (69.4) vs 2276 (69.8)	1035 (31.7) vs 1046 (32.1)	Empagliflozin 10 mg OD	Placebo	Hospitalization for HF, All-cause mortality, Cardiovascular mortality, All-cause hospitalization	26 months
(EMPACTMI)											
Von Lewinski *et al* 2022	Austria	421(212 vs 209)	57.7 ± 8.9 vs 58 ± 9.8	195 (82.3) vs 197 (82.42)	27.7 (25.3–30.3) vs 27.2 (24.9–30.2)	92 (39) vs 107 (45)	30 (13) vs 33 (14)	Empagliflozin 10 mg OD	Placebo	Hospitalization for HF, All-cause mortality, All-cause hospitalization, Mean change in LVEF	26 weeks
(EMMY Trial)											
James *et al* 2023	Sweden and United Kingdom	4017 (2019 vs 1998)	63.0 ± 11.06 vs 62.8 ± 10.64	1631 (80.8) vs 1579 (79.0)	-	-	-	Dapagliflozin 10 mg OD	Placebo	Hospitalization for HF, All-cause mortality, Cardiovascular mortality, All-cause hospitalization, Mean change in NT-proBNP difference	12 months
(DAPAMI)											
Shimizu *et al* 2020	Japan	96 (46 vs 50)	63.9 ± 10.4 vs 64.6 ± 11.6	38 (82.6) vs 39 (78.0)	25.2 (3.7) Vs 25.2 (4.1)	38 (82.6) vs 39 (78)	-	Empagliflozin 10 mg OD	Placebo	Hospitalization for HF, All-cause mortality, Cardiovascular mortality	24 weeks
(EMBODY)											
Lundin *et al* 2022	Sweden	42 (20 vs 22)	67.5 ± 8 vs 68 ± 8	16 (80.0) vs 18 (81.8)	27 ± 4 vs 27 ± 4	-	-	Empagliflozin 25 mg OD	Placebo	Mean change in NT-proBNP difference	10 months
(SOCOGAMI)											
Carberry *et al* 2024	United Kingdom	104 (51 vs 53)	63.4 ± 10.8 vs 62.6 ± 11.7	44 (86.3) vs 46 (86.8)	-	-	-	Empagliflozin 10 mg OD	Placebo	All-cause mortality, Mean change in NT-proBNP difference, Mean change in LVEF	24 weeks
(EMPRESSMI)											
Dayem *et al* 2023	Egypt	100 (50 vs 50)	55.24 ± 13.2 vs 56.70 ± 11.5	42 (84.0) vs 41 (82.0)	-	32 (64.0) vs 29 (58.0)	-	Dapagliflozin 10 mg OD	Placebo	Hospitalization for HF, All-cause mortality, Cardiovascular mortality, Mean change in LVEF	3 months
(DACAMI)											

^a^ Study population includes patients over the age of 18 with confirmed acute myocardial infarction

^b^ Data reported as mean ± SD

^c^ 22 countries including United States, Australia, Argentina, Brazil, Bulgaria, Canada, China, Denmark, France, Germany, Hungary, India, Israel, Japan, Republic of Korea, Netherlands, Poland, Romania, Russian Federation, Serbia, Spain, and Ukraine

### Risk of bias in included studies

**Figure 2.**
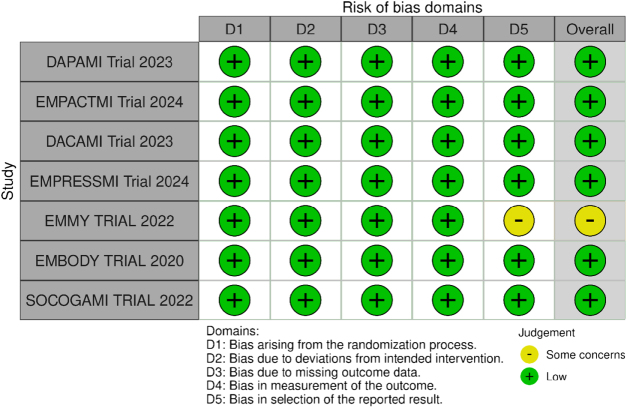
Risk of bias assessment of included studies.

The quality assessment of the included studies is presented in Fig. 2. Out of seven studies, six were deemed to have a low risk of bias across all assessed domains^[23–25,29,31,32]^. One study raised some concerns due to bias in the domain of selection of reported results^[30]^.

### Synthesis of results

#### Primary outcomes

##### Hospitalization for HF

**Figure 3.**
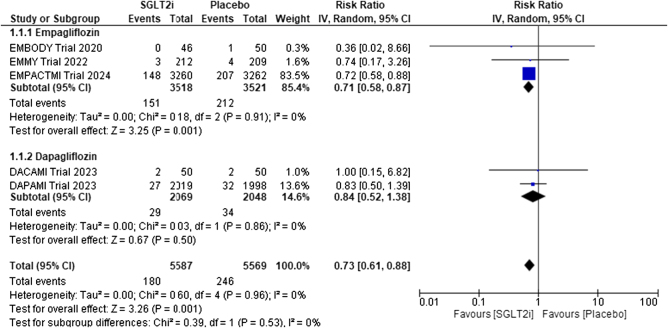
Forrest plot for hospitalization for HF.

Five studies were included in the pooled analysis to assess the risk of hospitalization for HF in patients using SGLT2 inhibitors compared to placebo. As shown in Fig. 3, SGLT2 inhibitors significantly reduced the risk of hospitalization for HF compared to placebo (RR: 0.73; 95% CI: 0.61, 0.88). No significant heterogeneity was reported among the study results (*I*^2^ = 0%). The absolute risk ratio (ARR) was 1.2% and the number needed to treat (NNT) was 83.6. On subgroup analysis based on the type of SGLT2 inhibitor, there was no statistically significant difference between the empagliflozin and dapagliflozin groups (*P* = 0.53).

##### All-cause mortality

**Figure 4.**
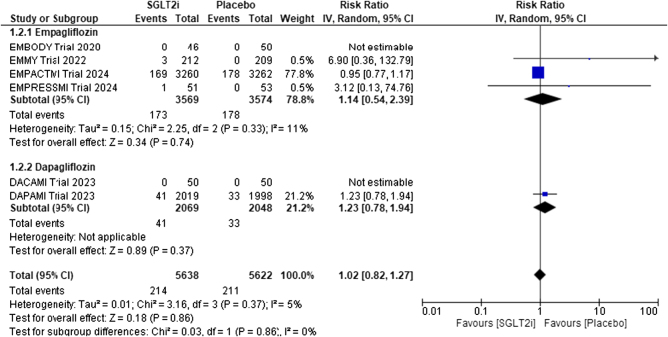
Forrest plot for all-cause mortality.

Six studies compared the risk of all-cause mortality. The pooled analysis indicated no statistically significant difference in all-cause mortality between the two groups. (RR, 1.02; 95% CI: 0.82-1.27) (Fig. 4). Minimal heterogeneity was reported among the study results (*I*^2^ = 5%). The absolute risk increase (ARI) was 0.0007 (95% CI; −0.0096 to 0.0064), and the number needed to harm (NNH) was 1406. We found no subgroup difference between empagliflozin and dapagliflozin regarding subgroup analysis for all-cause mortality (*P* = 0.85).

#### Secondary outcomes

##### Cardiovascular mortality

Four studies reported cardiovascular mortality and we found no statistically significant difference in the incidence of cardiovascular mortality in the SGLT2 inhibitors group as compared to the placebo group [Four RCTs, 10 735 patients (RR, 1.03; 95% CI, 0.83–1.28, *P* = 0.79)] (Supplementary Digital content, Figure S1, available at: http://links.lww.com/MS9/A928). The statistical heterogeneity was reported as minimal (*I*^2^ = 0%).

##### All-cause hospitalization

The pooled analysis of three RCTs with 10 960 patients showed a comparable rate of all-cause hospitalization between the SGLT2 inhibitors group and the placebo group (RR, 1.00; 95% CI, 0.84–1.17, *P* = 0.96) (Supplementary Digital content, Figure S2, available at: http://links.lww.com/MS9/A928). The statistical heterogeneity was reported as substantial (*I*^2^ = 67%).

##### Hypoglycemia

Five RCTs with 7185 patients showed no significant difference between the two groups regarding the incidence of hypoglycemia (RR, 0.80; 95% CI, 0.21–2.97, *P* = 0.74) (Supplementary Digital content, Figure S3, available at: http://links.lww.com/MS9/A928).

##### Hepatic injury

Three RCTs with 7085 patients reported that both groups did not differ significantly regarding the incidence of hepatic injury (RR, 2.39; 95% CI, 0.76–7.53, *P* = 0.14) (Supplementary Digital content, Figure S4, available at: http://links.lww.com/MS9/A928). The statistical heterogeneity was reported as minimal (*I*^2^ = 0%).

##### Ketoacidosis

The incidence of ketoacidosis was reported by two RCTs with 6995 patients. We found no significant difference between the two groups regarding the incidence of ketoacidosis (RR, 2.00; 95% CI, 0.18–22.01, *P* = 0.57) (Supplementary Digital content, Figure S5, available at: http://links.lww.com/MS9/A928).

##### Lower limb amputation

The pooled risk ratio of two RCTs with 6995 patients showed the incidence of lower limb amputation was comparable between the SGLT2 inhibitors group and the placebo. (RR, 1.80; 95% CI, 0.60–5.36, *P* = 0.29). (Supplementary Digital content, Figure S6, available at: http://links.lww.com/MS9/A928).

#### NT-pro BNP difference from baseline

N-terminal pro–B-type natriuretic peptide (NT-pro BNP) difference from baseline, a biomarker of cardiac stress and dysfunction, was reported in three trials. Raised NT-proBNP level from baseline with or without preserved ejection fraction has an adverse prognostic significance after AMI and is proven to be an independent predictive indicator of decompensated HF and cardiovascular death^[33]^. The analysis yielded a pooled mean difference of (MD, −0.23; 95% CI, −0.48 to 0.02) (Supplementary Digital content, Figure S7, available at: http://links.lww.com/MS9/A928), showing no significant reduction in NT-pro BNP level from baseline in the SGLT2 inhibitors group as compared to placebo with minimal statistical heterogeneity (*I*^2^ = 0%). This finding demonstrates no favorable impact of SGLT2 inhibitors on NT-pro BNP levels in patients after AMI.

#### LVEF at follow-up

Echocardiographic evidence of reduced LVEF was used to indicate impaired cardiac function and is crucial in measuring structural cardiac changes post-myocardial infarction. In two trials evaluating the LVEF at follow-up, patients in the SGLT2 inhibitors group showed no significant improvement in mean LVEF at follow-up when compared with the control group (MD, 0.27; 95% CI, −0.97 to 1.51) (Supplementary Digital content, Figure S8, available at: http://links.lww.com/MS9/A928). There was minimal statistical heterogeneity (*I*^2^ = 0%). This highlights no beneficial effects of SGLT2 inhibitors on improving LVEF following AMI.

## Discussion

In this systematic review and meta-analysis of seven randomized controlled trials comprising 11 302 patients, we meta-analyzed the safety and clinical efficacy of SGLT2 inhibitors in reducing mortality and HF in patients with AMI. We found that the institution of SGLT2 inhibitors led to a statistically significant reduction in hospitalization for HF following AMI compared to a matching placebo. The risk of all-cause mortality remained comparable between the two groups. Additionally, the pooled analysis maintained that the intended beneficial impact of SGLT2 inhibitors post-AMI on structural and functional cardiovascular parameters, including NT pro-BNP expression levels and LVEF, did not attain statistical significance.

The therapeutic benefits of SGLT2 inhibitors go beyond glycemic control. These inhibitors suppress sympathetic nervous activity and offer cardiovascular protection by reducing cardiovascular death and hospitalization for HF. This has been demonstrated in a systematic review by Shafaat Raza *et al*^[22]^, which aligns with the findings from the EMBODY trial included in our analysis^[31]^, further affirming the therapeutic efficacy and safety of SGLT2 inhibition. HF continued to be a frequent complication of AMI, reflecting the pathophysiological impact of myocardial insult at the cardiomyocyte and microvascular level^[34]^. Our clinical outcomes for hospitalization for HF and all-cause mortality parallel the largest trial, EMPACTMI^[24]^. However, this contrasts with the DAPAMI^[23]^ and other smaller trials regarding hospitalization for HF. This may be explained by the very small event rate as compared to the sample size, which may be catered by the inclusion of other larger RCTs in the future.

The reduction in hospitalization for HF with no effect on cardiovascular or all-cause mortality may be attributed to certain complications that occur during the first 30 days post-myocardial infarction. Various complications like recurrent myocardial infarction, mechanical complications, and scar-related ventricular arrhythmias may have contributed to mortality, thus nullifying the beneficial effect of SGLT2 inhibitors on mortality during this period^[35]^. The absence of significant mortality benefit in the acute setting may be due small event rate, as it may increase the risk of type II error^[36]^. Additionally, many trials were underpowered for mortality outcomes or lacked long-term follow-up, making definitive conclusions difficult, demanding a cautious interpretation of these findings. The timing of initiation of SGLT2 inhibitors is particularly important, as most of the studies added it toward the end of hospitalization (within 10 to 14 days of hospitalization), as in patients with type 2 diabetes, SGLT2 inhibitors are often withheld during the early days of hospitalization due to concerns about volume depletion, hypotension, and euglycemic ketoacidosis^[37,38]^. However, in the context of HF, especially with reduced ejection fraction, these agents are increasingly initiated or continued during hospitalization, supported by emerging trial evidence^[39]^.

The development of HF following AMI poses a substantial morbidity as well as mortality, which we have tried to assess. A recently published meta-analysis by Sinha *et al* evidenced the positive impact of SGLT2 inhibitor administration in AMI by curbing the risk of hospitalization for HF and cardiovascular mortality compared to placebo, with negligible effect on all-cause mortality^[21]^. Except for cardiovascular mortality, these findings align with our systematic review and meta-analysis. It should be kept in mind that most of the data in the aforementioned meta-analysis was observational in nature, with only one RCT and 8 cohort studies, which makes it prone to bias. Six newer RCTs have come to light since the aforementioned review, further strengthening the evidence.

Our findings for clinical parameters parallel the results of a recent meta-analysis by Mushood *et al*, which also showed a reduction in hospitalization for HF with no difference in cardiovascular or all-cause mortality^[20]^. The key differences between the systematic review of Mushood *et al* and our meta-analysis include the addition of SOCOGAMI and EMPRESSMI trials, both of which used Empagliflozin^[25,32]^. These additional RCTs further improved sample size and confidence in our estimates. The former meta-analysis lacked any data on the safety profile of SGLT2 inhibitors as well as functional and structural heart parameters like NT-pro BNP and LVEF, which are crucial for understanding the impact of SGLT2 inhibitors on cardiac remodeling post-myocardial infarction^[33]^. Unlike the former review, we did a subgroup analysis based on the type of SGLT2 inhibitors to ensure our findings remained consistent across the subgroups.

N-terminal pro–B-type natriuretic peptide (NT-pro BNP) is an important diagnostic and prognostic biomarker in patients with AMI, showing a strong correlation with cardiac stress and HF severity^[40]^. In animal studies, SGLT2 inhibitors appeared to mitigate myocardial oxidative stress and fibrosis, resulting in lower NT pro-BNP levels^[41,42]^, which is consistent with trial results by DACAMI^[29]^ and EMMY trial^[30]^. However, the SOCOGAMI and EMPRESSMI trial^[25,32]^ presented contrasting evidence, indicating no significant impact of SGLT2 inhibitors on reducing NT pro-BNP levels compared to placebo.

Cardiac remodeling following AMI involves a complex series of structural and functional changes in the heart^[33]^. Myocardial stunning may also play a role in transient systolic dysfunction post-myocardial infarction, which may improve after revascularization or lead to HF^[43]^. Echocardiographic evidence of reduced LVEF post-myocardial infarction signifies the deleterious effect of AMI on the left ventricle. SGLT2 inhibitors’ mechanism of action has a mechanistic link with cardiac parameters and is believed to impact echocardiographic parameters positively^[8]^. Previous trials established the favorable clinical effect of canagliflozin in improving the structural and functional parameters^[44,45]^, expressed by echocardiography, which parallel the findings of DACAMI^[29]^ and EMMY Trials^[30]^, though contrasting with the SOCOGAMI and EMPRESSMI trials^[25,32]^ results. However, our meta-analysis inferences appeared to contrast with no promising influence in reducing NT pro-BNP levels compared to placebo. We also failed to find any improvement in LVEF at follow-up, indicating no beneficial effect of SGLT2 inhibitors on structural heart parameters. This may be attributed to shorter follow-up times and smaller sample sizes among these trials

Despite providing concrete evidence from the latest RCTs, it is crucial to appraise the limitations of this meta-analysis. The length of follow-up in RCTs was not long enough to empower and consolidate the real-life clinical efficacy and safety profile of SGLT2 inhibitors in patients with AMI. Moreover, the included RCTs exhibited significant heterogeneity in respective trial protocols, including variability in age and geographical regions, prior co-morbidities, diabetic status, the length of follow-up, and duration of administration, the choice of SGLT2 inhibitor, pre-mature cessation of trial regimen^[24]^, and a change of methodological approach in the middle of the trial^[23]^. Hence, caution should be exercised while interpreting the comparisons, as the residual confounding cannot be completely excluded. Therefore, further large-scale RCTs with extended follow-ups, diverse geographical and racial representation, and overall well-powered protocols are needed to establish the real-life safety and clinical efficacy of SGLT2 inhibitors in this population.

To our knowledge, this is the first systematic review and meta-analysis to provide comprehensive insights into the safety and efficacy profile of SGLT2 inhibitors, specifically regarding their impact on structural and functional cardiac parameters in patients with AMI. SGLT2 inhibitors in reducing hospitalization for HF in patients presenting with AMI; however, the findings of the included RCTs did not exhibit statistical significance regarding mortality. This contradicts some of the findings observed across the HF population as discussed earlier^[39]^. Their diuretic effect, coupled with decreased metabolic demand, helps this particular population^[39]^. Thus, a more aggressive approach may be utilized across patients at a higher risk of HF (e.g., prior history of MI, old age, diabetes, etc.) post-AMI as compared to the low-risk population, where the mortality benefit may not be as pronounced^[46]^. The exact timing and particular subgroups may be further validated from the results of current ongoing trials, including DAPA-Protector, PRESTIGE AMI, and SGLT2-I AMI PROTECT Study probing the effect of SGLT2 inhibitors on cardiac structural and functional parameters following AMI^[47–49]^.

## Conclusion

In conclusion, while SGLT2 inhibitors show promise in reducing hospitalization for HF post-AMI, their impact on mortality and post-myocardial remodeling necessitates further investigation. This underscores the need for larger, more diverse RCTs to fully illustrate their role and timing of initiation in AMI management. An individualized approach based on risk assessment should guide their use in the post-AMI population.

## Data Availability

Data will be available from the authors on request.
